# Lithium treatment and human hippocampal neurogenesis

**DOI:** 10.1038/s41398-021-01695-y

**Published:** 2021-10-30

**Authors:** Alish B. Palmos, Rodrigo R. R. Duarte, Demelza M. Smeeth, Erin C. Hedges, Douglas F. Nixon, Sandrine Thuret, Timothy R. Powell

**Affiliations:** 1 Social, Genetic & Developmental Psychiatry Centre, Institute of Psychiatry, Psychology & Neuroscience, King’s College London, London, UK; 2 Department of Medicine, Weill Cornell Medical College, Cornell University, New York, NY USA; 3 Basic and Clinical Neuroscience, Institute of Psychiatry, Psychology & Neuroscience, King’s College London, London, UK; 4 Department of Neurology, University Hospital Carl Gustav Carus, Technische Universität Dresden, Dresden, Germany

**Keywords:** Molecular neuroscience, Stem cells, Pharmacology

## Abstract

Lithium is a first-line treatment for bipolar disorder, where it acts as a mood-stabilizing agent. Although its precise mechanism remains unclear, neuroimaging studies have shown that lithium accumulates in the hippocampus and that chronic use amongst bipolar disorder patients is associated with larger hippocampal volumes. Here, we tested the chronic effects of low (0.75 mM) and high (2.25 mM) doses of lithium on human hippocampal progenitor cells and used immunocytochemistry to investigate the effects of lithium on cell parameters implicated in neurogenesis. Corresponding RNA-sequencing and gene-set enrichment analyses were used to evaluate whether genes affected by lithium in our model overlap with those regulating the volume of specific layers of the dentate gyrus. We observed that high-dose lithium treatment in human hippocampal progenitors increased the generation of neuroblasts (*P* ≤ 0.01), neurons (*P* ≤ 0.01), and glia (*P* ≤ 0.001), alongside the expression of genes, which regulate the volume of the molecular layer of the dentate gyrus. This study provides empirical support that adult hippocampal neurogenesis and gliogenesis are mechanisms that could contribute to the effects of lithium on human hippocampal volume.

## Introduction

Lithium has been used in the treatment of bipolar disorder symptoms for over 50 years [1]. Despite its longevity as a mood stabilizer, questions remain regarding its effects on the brain and its therapeutic mechanism of action. Recent neuroimaging studies, implicate the hippocampus as one important target of lithium, with both mouse [2] and human [3] studies showing an accumulation of lithium in the hippocampal regions of the brain following repeated oral administration.

The hippocampus is a brain area, which regulates learning, memory, cognition, and mood [4], and large mega-analyses from the ENIGMA neuroimaging consortium suggest smaller hippocampal volumes are a common feature amongst psychiatric disorder patients [5–7]. Amongst bipolar disorder patients, however, chronic lithium users exhibit larger hippocampal volumes relative to their peers who are either unmedicated or undergoing other forms of treatment [8]. This suggests that lithium treatment may normalize hippocampal volume reductions observed in psychiatric disorder patients, either by protecting existing neurons [9], or by promoting neurogenesis [10].

Unlike most of the brain, the mammalian hippocampus is capable of neurogenesis even in adulthood [11], with estimates of up to 700 new neurons being generated each day in the human dentate gyrus [12]. Environmental [13], genetic [14] and pharmacological factors [15] can influence the rate of neurogenesis in the adult hippocampus, either by affecting the rate of cell proliferation along the subgranular zone of the dentate gyrus, or the rate at which these cells differentiate into neurons and glia. In the context of lithium, it has been hypothesized that part of its mechanism of action involves triggering neurogenesis in the dentate gyrus, which would subsequently increase the density of neurons (and glia) and contribute to the observed increase in hippocampal volume and improvement in mood and cognition [16]. Indeed, the neurogenic properties of lithium have been reported previously in animal models, whereby lithium either increases the number of proliferating [10, 17, 18] or differentiating neural cells [19]. However, there has been a marked absence of studies investigating the effects of lithium on human hippocampal cells. There is also no empirical data connecting the neurogenic effects of lithium observed in model systems to the effects observed in the human brain (i.e., from neuroimaging data). The current study aimed to bridge this gap, and to provide empirical evidence either supporting or refuting the relationship between lithium, human hippocampal neurogenesis, and hippocampal volume.

Here, we employed an in vitro protocol [20, 21] to assess the chronic effects of low (0.75 mM) and high (2.25 mM) doses of lithium on human hippocampal progenitor cells, assessing effects on global gene expression, cell proliferation, and cell differentiation. We found a significant increase in the generation of neuroblasts, neurons, and glia in response to high-dose lithium, with no changes in the rates of cell proliferation or cell death. Corresponding RNA-sequencing (RNA-seq) data and gene ontology (GO) enrichment revealed a change in genes responsible for neuronal development and the extracellular matrix, in response to high-dose lithium. Gene-set enrichment analyses were used to test whether genes affected by lithium overlap with those regulating the volume of distinct layers of the dentate gyrus. We found that genes upregulated in response to high-dose lithium are specifically enriched for variants associated with the volume of the molecular layer of the dentate gyrus, where the majority of dendritic branching occurs amongst adult-born cells. Collectively, our work suggests that chronic lithium treatment increases human hippocampal progenitor differentiation, and by doing so, increases the volume of the molecular layer of the dentate gyrus and subsequently total hippocampal volume.

## Methods

### Human hippocampal progenitor cell line

An existing multipotent human hippocampal progenitor cell line, HPCOA07/03 (ReNeuron, UK), was used to model human hippocampal neurogenesis in vitro, as used previously by our team [20–23]. The cell line was derived from first-trimester, female, foetal hippocampal tissue following medical termination, and in accordance with UK and USA ethical and legal guidelines, and obtained from Advanced Bioscience Resources (Alameda CA, USA). These cells proliferate in the presence of growth factors, and upon their removal, differentiate into hippocampal PROX-1-positive cells, MAP-2-positive neurons, and S100β-positive glia. For further details on the cell line and culture conditions for proliferating and differentiating cells, see Supplemental Information, S1–S2.

### Chronic lithium treatment

**Fig. 1:**
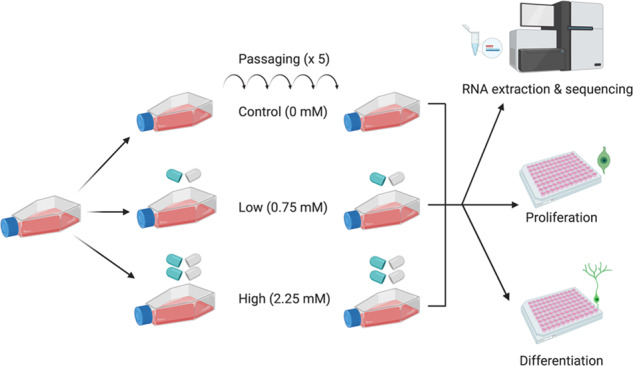
A summary of our experimental protocol. Cells were treated with control (0 mM), low (0.75 mM) or high (2.25 mM) doses of lithium across five passages (~15 days) and submitted to nucleic acid extraction and RNA-sequencing, a proliferation protocol, and adifferentiation protocol.

Cells were treated chronically with either a vehicle control (0 mM), a therapeutically relevant low dose of lithium (0.75 mM) similar to what’s found in the blood of bipolar disorder patients [24], or a high dose of lithium (2.25 mM) similar to what’s been used in previous in vitro work [25]. Cells were grown in T75 flasks and were maintained in proliferating cell media and a constant lithium concentration across five passages (~15 days), see Supplemental Information S2. This duration was selected because it exceeds the minimal time period required to observe clinically meaningful effects of lithium in bipolar disorder patients [26]. Four biological replicates were utilized in order to detect medium-to-large effect sizes [27]. Following this, subsets of cells were either (i) lysed and submitted to nucleic acid extraction for subsequent RNA-sequencing, (ii) seeded on to a 96-well plate and submitted to a proliferation assay, followed by immunocytochemistry, or (iii) seeded on to a 96-well plate and submitted to a differentiation assay, followed by immunocytochemistry, Fig. 1.

### Proliferation and differentiation assays (Immunocytochemistry)

To compare differences in cell marker levels between control, low, and high lithium conditions, we performed immunocytochemistry using an established 3-day proliferation protocol performed on 96-well plates, where we assayed the proliferation markers BrdU and Ki67, and the apoptosis marker cleaved caspase-3 (CC3). We also assessed differentiation as part of a 10-day protocol, where we assayed the neuroblast marker doublecortin (DCX), the neuronal marker microtubule-associated protein 2 (MAP2), the glial marker S100β, and the apoptotic marker CC3. Cells were maintained in their respective lithium doses across both assays. See Supplemental Information, S3–S5 and Supplementary Table 1 for further details.

### RNA-sequencing

Total RNA was extracted using the Qiagen AllPrep DNA/RNA/Protein Mini Kit (Qiagen, Hilden, Germany). All RNA samples were of good purity and integrity, as indicated by 260/280 ratios of between 1.95 and 2.1, tested using a Nanodrop ND-1000 spectrophotometer (Thermo Scientific, Wilmington, DE), and RINs > 9, as tested using an Agilent 2100 Bioanalyzer (Agilent Technologies, Berkshire, UK). Library preparation and RNA-sequencing were performed at The Genomic Centre, King’s College London. Briefly, total RNA samples were submitted to a DNase treatment using the DNA-free™ DNA Removal Kit (Invitrogen, California, USA). Subsequently, 300 ng of total RNA from each sample was submitted for ribosomal RNA depletion using the NEBNext rRNA Depletion kit (New England Biolabs, Massachusetts, USA), and RNA-Seq libraries were constructed using the NEBNext Ultra II Directional RNA Library Prep Kit for Illumina (New England Biolabs). The samples were sequenced in a HiSeq 4000 sequencing system (Illumina). Raw reads were downloaded and processed using a systematic approach, see Supplemental Information, S6. As the clearest effect on neurogenesis was observed between the control group (vehicle treated) versus the high-dose lithium group, our primary gene expression analysis was limited to these two groups. Differential expression analysis was achieved using Wald tests in DESeq2, controlling for biological replicates (*N* = 4 biological replicates per condition). Log2 fold-changes were shrunk using apeglm [28], and the false discovery rate (FDR) correction was used to control for multiple comparisons. As a secondary, complementary analysis, we also generated RNA-seq data comparing the control group (vehicle treated) versus the low therapeutically relevant dose of lithium, in order to establish whether effects in the high-dose group overlapped with those observed in the ‘therapeutically relevant’ low-dose condition. In accordance with the UK research councils’ Common Principles on Data Policy, RNA-seq data supporting this study is openly available via GEO, under the accession code GSE184930.

### Gene ontology (GO) enrichment

To understand which biological mechanisms were being affected in our cell model in response to high-dose lithium treatment, we separately entered genes (*P*_FDR_ < 0.05) showing an increase in expression, and those showing a decrease in expression, into WebGestalt [29]. We used the Overrepresentation analysis to identify relevant gene ontology terms corresponding to biological processes, cellular components, and molecular functions. We included all transcripts surviving DESeq2’s internal filtering criteria as our background list (i.e., all genes expressed in the cell line).

### Gene-set enrichment analysis

We tested for a genetic overlap between upregulated and downregulated genes affected by high-dose lithium in our model, and those regulating the volume of the whole hippocampus, or distinct layers of the dentate gyrus (where neurogenesis takes place), including the hilus, the granular cell layer, and the molecular cell layer. We achieved this by utilizing publicly available summary statistics from a GWAS that used neuroimaging methods to estimate the volume of hippocampal subfields in over 21,000 individuals [30]. Within this study, the volume of each layer of the dentate gyrus was corrected for total hippocampal volume in order to isolate genetic effects specific to each layer. Gene-level followed by gene-set enrichment analysis was performed in MAGMA [31] after removing the region encompassing the major histocompatibility complex (chromosome 6, 25–34 Mb), due to the complex linkage disequilibrium structure of this locus. We used a 10 kb window around genes to link the polymorphisms tested in the GWAS with all established protein-coding genes in the genome. The gene-level enrichment was calculated by generating a gene-wide statistic from the GWAS summary statistics, adjusting associations for gene size, variant density, and linkage disequilibrium using the 1000 Genomes Phase 3 European reference panel. Subsequently, we used MAGMA to perform a competitive test to calculate the enrichment of how over-represented the genes in our gene sets (up- or downregulated genes in the cell model) were in relation to those implicated in each trait (i.e., hippocampal volume), relative to other gene sets of similar size across the genome.

### Statistical analyses

ANOVA followed by Tukey’s multiple comparison tests were used to assess the effects of lithium on cell markers. The normality of cell marker data was confirmed using the Shapiro-Wilk test. RNA-sequencing data and downstream analyses were performed as described above. The False Discovery Rate (FDR) method of multiple testing correction was applied to each analysis, where we considered an *P*_FDR_ < 0.1 as significant. For the RNA-sequencing analysis we applied a more conservative threshold (*P*_FDR_ < 0.05) in order to derive gene sets comprising of fewer than 500 genes for the gene-set enrichment analysis.

### Figure generation

Figures were created using Prism7 (GraphPad, San Diego USA) and BioRender (Toronto, Canada). The volcano plots were generated using WebGestalt [29]. The gene network figure was generated using GeneMANIA [32].

## Results

### Lithium does not affect rates of cell proliferation relative to vehicle control

We analysed hippocampal progenitor cells chronically treated with high/low/no lithium using a screen of cellular markers, and estimated the percentage of cells expressing markers of neural stem cell proliferation and programmed cell death. ANOVA revealed no significant differences between the vehicle control and lithium-treated samples in relation to the proliferation marker BrdU (*F*(2, 9) = 0.367, *p* = 0.703), suggesting that the most recent levels of proliferation were unaffected by the low and high lithium doses. Results pertaining to the apoptotic marker CC3 (*F*(2, 9) = 0.735, *p* = 0.506) indicate that the lithium doses used in our experiment were not inducing cell death and thus were unlikely to be toxic.

**Fig. 2:**
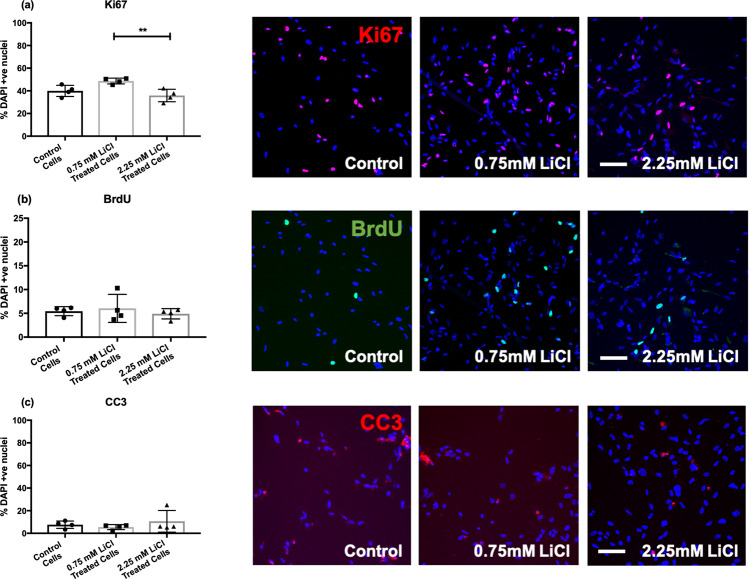
Bar charts showing the percentage of Ki67, BrdU, and CC3 positive cells in those treated chronically with a vehicle control (0 mM), low (0.75 mM), or high (2.25 mM) dose of lithium chloride. The bar charts (left) show the percentage of Ki67 (**a**), BrdU (**b**), and CC3 (**c**) positive cells relative to the percentage of DAPI stained nuclei in control and lithium-treated cells. Bar heights correspond to means, and the standard errors of the mean (S.E.M.) are indicated. Each data point represents one biological replicate (*N* = 4). * represents an uncorrected *P* ≤ 0.05. ** represents an uncorrected *P* ≤ 0.01. *** represents an uncorrected *P* ≤ 0.001. Each of the images (right) are representative of a field of immuno-stained cells, taken using a 10X objective with the CellInsight High Content Screening Platform. Each image includes the nuclear marker DAPI in blue. Scale bar = 100 μm.

ANOVA did show a significant difference in relation to the proliferation marker Ki67 (*F*(2, 9) = 8.384, *p* = 0.009, *P*_FDR_ < 0.1), which unlike BrdU captures differences in proliferation over a greater time frame. However, the effect was only observed between the low and high-dose lithium conditions (*p* = 0.008), where levels of proliferation were highest in the low-dose condition. There were no significant effects observed relative to the vehicle control (*p* > 0.05), Fig. 2.

### High-dose lithium increases rates of hippocampal progenitor cell differentiation

**Fig. 3:**
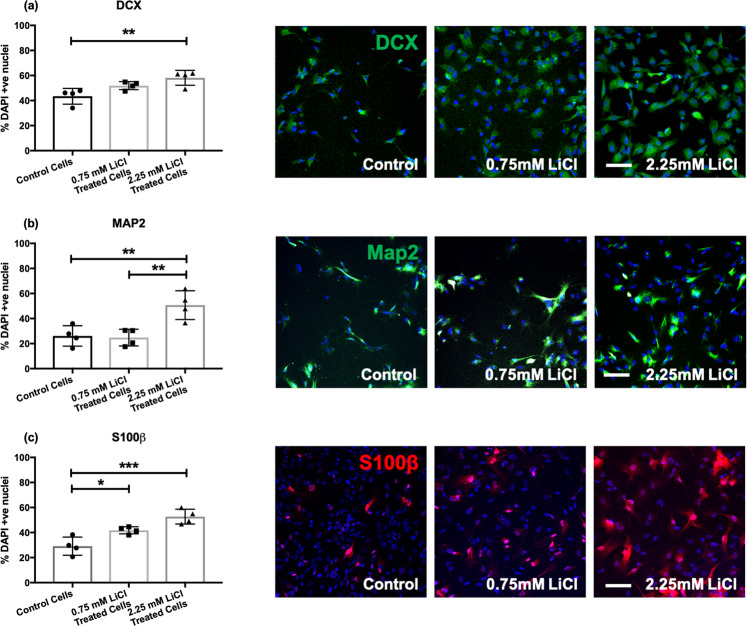
Bar charts showing the percentage of DCX, MAP2, and S100β positive cells in those treated chronically with a vehicle control (0 mM), low (0.75 mM), or high (2.25 mM) dose of lithium chloride. The bar charts (left) show the percentage of DCX (**a**), MAP2 (**b**), and S100β (**c**) positive cells relative to the percentage of DAPI stained nuclei in control and lithium-treated cells. Each data point represents one biological replicate (*N* = 4). * represents *P* ≤ 0.05, ** represents *P* ≤ 0.01, and *** represents *P* ≤ 0.001, based on the result of Tukey’s post hoc tests. Each of the images (right) are representative of a field of immuno-stained cells, taken using a 10X objective with the CellInsight High Content Screening Platform. Each image includes the nuclear marker DAPI in blue. Scale bar = 100 μm.

We analysed the effects of lithium on markers of neurons and glia, whilst cells were differentiating. ANOVA revealed significant differences between lithium-treated cells and the vehicle control, in relation to the percentage of DCX-positive neuroblasts (*F*(2, 9) = 7.616, *P* = 0.012), the percentage of cells expressing the neuronal marker MAP2 (*F*(2, 9) = 10.55, *p* = 0.004) and the percentage of cells expressing the glial marker, S100β (*F*(2, 9) = 17.48, *p* = 8.000 × 10^–4^). All effects on neural cell differentiation remained significant after correcting for the number of markers tested (*P*_FDR_ < 0.1). In all comparisons, high-dose lithium increased rates of cell differentiation relative to vehicle-treated control cells (*p* < 0.01), Fig. 3. We additionally stained for the apoptotic marker CC3, and found consistently low levels of cell death (<5%) which did not differ between conditions (*F*(2, 9) = 0.774, *p* = 0.490).

### High-dose lithium exerts genome-wide expression changes in human hippocampal progenitor cells related to nervous system development and the extracellular matrix

**Fig. 4:**
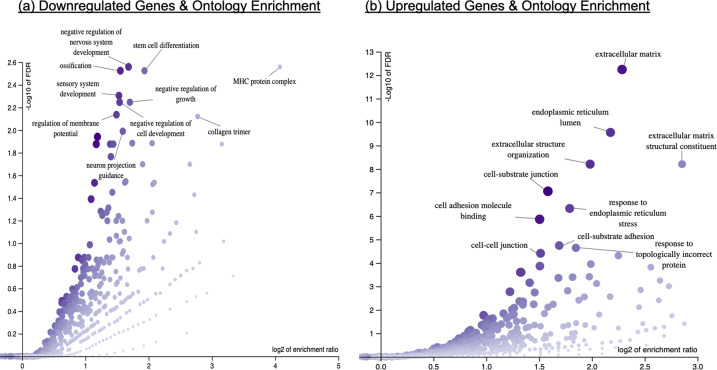
Volcano plots illustrating significantly enriched gene ontology terms in response to high-dose lithium. **a** GO terms significantly downregulated in response to high-dose lithium, (**b**) GO terms significantly upregulated in response to high-dose lithium.

The high-dose lithium condition was associated with the differential expression of 710 genes (*P*_FDR_ < 0.05), relative to cells treated with the vehicle control, see Supplementary Dataset 1 for full results. The 327 genes downregulated in our model were enriched for GO terms related to nervous system development and the MHC protein complex. The 383 upregulated genes were enriched for GO terms related to cell adhesion, the extracellular matrix, and endoplasmic reticulum, Fig. 4. Whilst the effect of lose dose lithium on gene expression was more subtle, approximately one-third of the genes nominally affected by this dose (*P* < 0.05), were also affected by the high-dose condition (*P*_FDR_ < 0.05; 78 out of 244 genes), but with a stronger magnitude of effect (log_2_ fold change), see Supplementary Datasets for full results.

We also considered how our gene expression results compared to genes previously implicated in bipolar disorder and lithium response. Out of 45 candidates previously identified [33] only two were significantly affected in our model—Protein kinase B (*AKT1*) and Myristoylated alanine-rich C-kinase substrate (*MARCKS*) (*P*_FDR_ < 0.1), both of which were downregulated in response to high-dose lithium. See Supplemental Information, S7 and Supplemental Fig. 1, for further information on these results and a discussion of these candidates.

### Genes upregulated in response to high-dose lithium also regulate the volume of the dentate gyrus

**Fig. 5:**
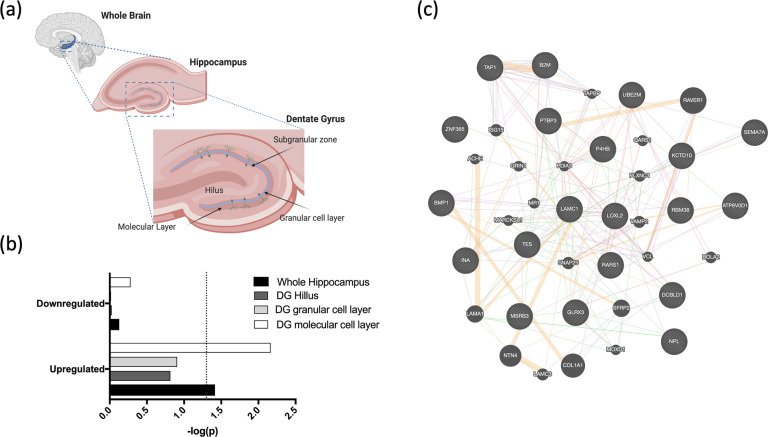
Lithium’s effect on the expression of genes in hippocampal progenitors and its overlap with genes regulating the volume of the dentate gyrus. **a** The anatomy of the hippocampus, including the dentate gyrus where adult neurogenesis occurs. **b** A bar chart illustrating the results from our gene-set enrichment analyses. Gene sets comprise of upregulated and downregulated genes (*P*_FDR_ < 0.05) in response to the high lithium dose. These sets of genes were tested for overlap with those implicated in the regulation of whole hippocampal volume and the volume of dentate gyrus (DG) subfields where neurogenesis takes place. Stronger significance is indicated by a larger bar, and -log(*p*) value. The dashed line represents the nominal significance threshold (*p* = 0.05). The overlap between genes upregulated in response to lithium and those regulating the size of the molecular cell layer of the dentate gyrus was the only test that remained significant after multiple testing correction (*P*_FDR_ < 0.1). **c** A gene network image generated using GeneMANIA. This describes genes significantly affected by lithium in our hippocampal progenitor cell model (*P*_FDR_ < 0.05), which were also associated with volume in the molecular layer based on gene-level enrichment analyses (*P* < 0.05). Lines represent expected interactions between genes (orange = predicted interactions, purple = interactions based on co-expression, pink = physical interactions, blue = interactions based on colocalization, green = genetic interactions, yellow = shared protein domains). Genes input into GeneMANIA are represented by circles with diagonal lines, genes hypothetically interacting within this network are represented by solid circles. *LAMC1* interacted with the most genes relative to other genes input into GeneMANIA, suggesting it might represent an important hub gene.

Our gene-set enrichment analyses revealed a nominal overlap between genes upregulated in response to lithium and those regulating whole hippocampal volume (*β* = 0.072, SE = 0.041, *p* = 0.03). We then tested for overlaps with subfields of the dentate gyrus (where neurogenesis occurs), and found a specific overlap between the genes upregulated in response to high-dose lithium and those associated with the volume of the molecular layer of the dentate gyrus (*β* = 0.100, SE = 0.041, *p* = 0.007, *P*_FDR_ < 0.1), Fig. 5b. To better understand which genes/networks within our gene set contributed to this positive relationship, we identified 20 genes that were significantly affected in our model (*P*_FDR_ < 0.05) that were also at least nominally associated with volume in the molecular layer (*P* < 0.05) based on the gene-level enrichment analysis. We then performed gene network analysis using GeneMANIA to consider how these genes relate to one another. This revealed a significant enrichment for collagen-containing extracellular matrix genes (*P*_FDR_ < 0.05), and identified Laminin Subunit Gamma 1 (*LAMC1*) as a potential hub gene, because it interacted with 9 of the other significant genes within that network (more than any other gene), Fig. 5c.

## Discussion

Lithium accumulates in the neurogenic regions of the brain and exerts a positive effect on hippocampal volume in bipolar disorder patients [2, 3, 8]. Given the existence of the neurogenic niche in the dentate gyrus of the hippocampus, we tested the chronic effects of lithium treatment on parameters of neurogenesis in human hippocampal progenitor cells, and used population genetic methods to infer their putative relationship with adult hippocampal volume.

As opposed to non-human animal work [9, 10, 17, 18, 34], our results generally did not show an effect of lithium on markers of cell proliferation (BrDU, Ki67) or programmed cell death (CC3). The absence of effects on the expression of these markers may represent a species-specific difference [35], or reflect the specific treatment duration utilized in our model. Alternatively, it may be that lithium preferentially protects mature neurons as opposed to neural progenitor cells, or cells that are challenged with a stressor. Previous research has demonstrated neuroprotective effects of lithium in response to cellular stress, inflammation, or genetic perturbations (e.g. amyloid precursor protein upregulation), and thus the absence of a stressor (or specific genetic driver) in our model may explain the absence of clear effects [36]. Curiously, we did observe increased proliferation in the low-dose lithium group relative to the high-dose group (marked by Ki67); however, neither condition was significantly different from the control, Fig. 2. It is possible that a low-dose lithium treatment exerts a subtle increase in proliferation, whereas high doses drive cells away from proliferation and toward differentiation. Further work in independent model systems and larger sample numbers may be useful in teasing apart more subtle dose-specific effects of lithium on cell proliferation.

Our immunostaining results did reveal a clear positive effect of chronic high-dose lithium on human hippocampal progenitor cell differentiation, with increases observed in the percentage of cells expressing both neuronal and glial markers. This supports animal work which shows that lithium can increase hippocampal cell differentiation by increasing the percentage of MAP2-positive cells [19]. Although the mechanism via which lithium exerts its effects on cell differentiation will require further research, our RNA-sequencing data hints at early transcriptomic changes to proliferating cells which might facilitate subsequent cell differentiation. For instance, amongst proliferating cells treated with high-dose lithium, we observed an upregulation of genes responsible for cell adhesion and the extracellular matrix. The extracellular matrix, a three-dimensional collection of proteins that surrounds cells, provides biochemical and structural support for cells as they develop [37]. It exerts direct effects on neural cell differentiation, axon development, and myelination [38], and thus the increase in expression of extracellular matrix genes whilst cells are proliferating may provide an environment that better facilitates differentiation, which could ultimately facilitate neuroplasticity in the hippocampus. This is further supported by recent work revealing the effects of lithium on both the extracellular matrix and on astrocyte morphology [39]. Similarly, cell adhesion is a process that enables neurite outgrowth, cell migration, axon guidance, and synaptogenesis, which also plays a critical role in neuroplasticity [40].

To contextualise our in vitro findings further, we should consider how our results could potentially impact the structure and function of the dentate gyrus, in vivo. The dentate gyrus is the site of neurogenic activity in the hippocampus and consists of distinct layers, each providing different niches for cell growth and development [41]. The subgranular zone houses proliferating neural progenitor cells and it is the site of adult neurogenesis [42]. As progenitor cells differentiate and specialize into neurons, they generate dendrites, which project outwards through the granular cell layer and into the molecular layer, where they expand to form new dendritic branches (with the aid of cell adhesion molecules and proteins in the extracellular matrix), Fig. 5. Axons also extend inwards, towards the hilus where they form synaptic connections with unmyelinated axons (mossy cells) and integrate into the pre-existing neuronal circuitry [41]. Our work reveals a specific impact of lithium on neural cell differentiation, and we can infer from its effects on gene expression that it is likely to specifically impact on the volume of the molecular layer of the dentate gyrus. Based on these findings, we theorize that lithium triggers early transcriptomic changes to progenitor cells that increase their rate of differentiation, ultimately leading to the expansion of cells into the molecular layer. As the molecular layer represents the outermost layer of the dentate gyrus and this is the area where neurons are branching outward, it is possible that increased neural cell differentiation (and density) would consume space within this layer, and that this would subsequently force the hippocampus to expand in volume.

To narrow down which genes might link lithium’s effect in our cell model, to the volume of the molecular layer, we isolated the genetic overlap and performed gene network analysis. Similarly to the effect of lithium in our model using all genes surpassing the multiple correction threshold, this subset was enriched for genes related to the collagen-containing extracellular matrix, Fig. 5c. At its hub was Laminin Subunit Gamma 1 (*LAMC1*), which is an extracellular matrix glycoprotein that plays important roles in cell adhesion, differentiation, and neurite outgrowth [43]. Future research should consider whether *LAMC1* and the network of genes it interacts with could be used to inform alternative ways of promoting neurogenesis and hippocampal volume in psychiatric disorder patients.

Given the relatively small size of the dentate gyrus, it is possible that lithium affects cell mechanisms outside of this region too, which could contribute to overall volume differences observed amongst chronic lithium users. For instance, the strongest effect we observed in our cell model was lithium’s impact on gliogenesis. Unlike neurons, new glia continue to form in the hippocampus, and across the brain, throughout life, due to the widespread presence of glial progenitor cells [44]. Consequently, an increase in glial cell differentiation could also contribute to volume changes observed in bipolar disorder patients treated with lithium. Not only could an increased number of glial cells consume more space within the hippocampus, these cells also facilitate the remyelination of neurons, which could contribute to structural and functional changes leading to volume increases.

Despite the insights discussed here, there are limitations of our study that should be acknowledged. First, the effects of lithium are known to be moderated by genetic factors [45], and so by using a single cell line we may be missing important information relating to its mechanism of action in a broader context. Second, our gene expression data was collected in proliferating cells, whereas the clearest effect of lithium on neurogenesis-related markers was observed once these cells were differentiated. Consequently, we may be missing important effects of lithium on the transcriptome whilst cells are differentiating. However, previous work using antidepressants in this cell line demonstrated that drug treatments whilst cells are proliferating are actually more pivotal in fast-tracking downstream increases in cell differentiation [46]. This effect is likely mediated by early gene expression changes to proliferating cells (i.e., those captured in our RNA-seq data) that prime cells to more readily differentiate into adult cells [46]. Third, we used population genetic methods to bridge the gap between what we observed in human cells in vitro, and what is observed in neuroimaging studies. Although this sort of approach is novel and important, it is still inferential. Future studies should verify that lithium exerts an effect on hippocampal volume via the molecular layer of the dentate gyrus, by observing changes in this hippocampal subfield in patients treated with lithium longitudinally. Fourth, we observed the majority of lithium’s effects in the high-dose condition which although are consistent with concentrations used in other in vitro work [47], may not be representative of the concentration found in the blood of patients or their hippocampus. Nonetheless, directly extrapolating drug doses from an in vivo experiment to an in vitro one is complicated [48]. Whilst we acknowledge that the high dose of lithium could exert toxic effects in patients (e.g., in the thyroid and kidneys), the immunocytochemistry and gene expression results do not reveal any impact of high-dose lithium on markers of cell death (e.g., no effects on CC3 levels) in our hippocampal model. The RNA-seq results also reveal an overlapping effect of what is observed in response to the low and highdose conditions, supporting the notion that high-dose lithium affects similar molecular mechanisms as the ‘therapeutically relevant’ low-dose in our model, but with a stronger magnitude of effect. It is possible that a longer treatment duration with the low dose would more closely recapitulate the effects of the higher dose used in our protocol. Finally, our study makes use of a conditionally immortalized, fetal-derived cell line and thus the effects observed may not reflect what occurs in adult progenitor cells in vivo. Differentiation of induced pluripotent stem cells into hippocampal cultures offers an alternative way to validate findings reported here, and to consider lithium’s effects in cells from psychiatric disorder patients [49]. Alternatively, in vivo models with mature cells and established intercellular networks could provide even greater construct validity [50] that are not possible from tissue culture experiments.

To conclude, this study reveals a positive effect of chronic high-dose lithium on human hippocampal progenitor cell differentiation which correlates with an upregulation of genes that mediate cell adhesion or constitute the extracellular matrix, as well as those that regulate the volume of the molecular layer of the dentate gyrus. Our research suggests that adult neurogenesis and gliogenesis could be mechanisms contributing to lithium’s effect on hippocampal volume. Future research should continue to bridge the gap between in vitro and in vivo observations, and to explore whether neurogenesis is also a critical mechanism explaining lithium’s therapeutic effects.

## Supplementary information

Supplemental Information

Supplementary Dataset
