# Supplementary material for: Lithium treatment and human hippocampal neurogenesis
Source: Transl Psychiatry. 2021 Oct 30;11:555. doi: 10.1038/s41398-021-01695-y (PMC8557207; doi:10.1038/s41398-021-01695-y)
Supplement: Supplementary file 1 — Supplemental Information [file 41398_2021_1695_MOESM1_ESM.docx]

**Supplemental Information**

**S1: Hippocampal Progenitor Cell Line**

We utilised a multipotent human foetal hippocampal progenitor cell line (HPCOA07/03; ReNeuron, UK), as in our previous work ^1-4^. The cell line was derived from first-trimester, female, foetal hippocampal tissue following medical termination, and in accordance with UK and USA ethical and legal guidelines, and obtained from Advanced Bioscience Resources (Alameda CA, USA).

HPC0A07/03C cells were *conditionally* immortalised by introducing the c-Myc-ER^TAM^ transgene, whereby upon activation of the modified oestrogen receptor with 4-hydroxy-tamoxifen (4-OHT), the oncogene c-Myc becomes activated and maintains high levels of proliferation in culture, with the aid of the growth factors, epidermal growth factor (EGF) and basic fibroblast growth factor (bFGF)^5^. Upon removal of growth factors, cells differentiate into doublecortin (DCX)-positive immature neuroblasts, microtubule-associated protein 2 (MAP2)-positive neurons, and S100β-positive astrocytes. The remaining population maintain a neural progenitor cell phenotype. Immunocytochemistry has confirmed that MAP2-positive neurons co-stain for Prospero homeobox protein 1 (PROX1), which is a marker used to identify neurons from the dentate gyrus^2^.

**S2: Cell Culture conditions**

Cells were cultured in monolayer on Nunclon flasks (Thermo Fisher Scientific, Massachusetts, USA; #156367/156499), coated with 23 µg/ml laminin from Engelbreth–Holm–Swarm murine sarcoma basement membrane (Sigma, Missouri, USA) in phosphate buffered saline (Gibco, Massachusetts, USA; #L2020) for 1–24 hrs to aid cell adhesion. When passaging, media was aspirated and cells were exposed to warm Accutase (Sigma, #A1110501) to lift them from monolayer, they were then resuspended in warm medium, and washed twice via centrifugation at 900 rpm for 5 minutes with subsequent resuspension. Cells were maintained in chemically-defined proliferation medium and incubated at 37 °C, 5% CO_2_ and 100% humidity. Proliferation medium was composed of Dulbecco’s modified Eagle’s Medium Nutrient Mixture F-12 Ham (DMEM-F12; Sigma–Aldrich, #D6421 or #D6434) supplemented with 0.03% human albumin solution (Zenalb, #20), 100 μg/ml human apo-transferrin (Sigma, #T1147), 16.2 μg/mL human putrescine DiHCl (Sigma, #P5780), 5 μg/mL human recombinant insulin (Sigma, #I9278), 60 ng/mL progesterone (Sigma, #P8783), 2 mM l-glutamine (Sigma, #G7513) and 40 ng/mL sodium selenite (Sigma, #S9133). 10 ng/mL human bFGF (Peprotech, New Jersey, USA; #AF 100-15-500) and 20 ng/mL human EGF (Peprotech, #EC 100-18B) were including in proliferating cell media only. The cells were regularly checked (daily via light microscopy and twice via mycoplasma detection) to make sure they were growing without any infections. Whilst cells are usually expanded in the presence of 4-OHT, to reduce any confounding effects of c-Myc activation on gene expression profiling^6^, we eliminated 4-OHT upon reactivation of cells and grew them for four passages in the presence of just EFG and bFGF, as previously^7^, after which we initiated drug experiments.

Lithium Treatment: Lithium chloride (LiCl) was obtained from Sigma (Sigma, Gillingham, UK) and reconstituted in sterile phosphate buffered saline to produce a 1 M stock concentration in 50 ml. This was split into five 10 ml working volumes and stored at 4 ^O^C. One tube was used for each biological replicate and was used within six months of reconstitution. Relevant concentrations of LiCl were made by dilution in cell media and chosen to reflect a therapeutically relevant “low dose” (0.75 mM)^8^, and a “high dose” (2.25 mM) that falls within a range previously used in *in vitro* work^9^. Cell media was also used for the vehicle control (0 mM).

Cells were treated chronically with lithium in T75 flasks by maintaining their relevant treatment concentration for five passages. For each passage, cells were treated until they reached 80-90% confluency (~3 days). The differences in the length of treatment did not deviate significantly between lithium dose groups (P > 0.05). Cells were then either collected for nucleic acid extraction or submitted to proliferation and differentiation assays, where again they were maintained in the media corresponding to the same concentration of lithium.

**S3: Proliferation and differentiation assays**

Cells were seeded on laminin-coated 96-well plates at a density of 1.2 x 10^4^ cells/well in 100 μL of proliferating cell medium. Three technical replicates (wells) were generated in relation to each staining marker, alongside a negative staining control (no primary antibodies). Proliferating medium was aspirated 24 hrs after seeding and replaced with fresh medium. Cells were then grown for 48 hrs, after which they were fixed using a 4% paraformaldehyde solution. For three of the wells, we included a Bromodeoxyuridine (BrdU) incorporation step, consisting of a 10 μM BrdU treatment for the last 4 hrs prior to fixing. Subsequently, immunocytochemistry was used to assay proliferation markers including BrdU and Ki67, and the apoptosis marker caspase-3 (CC3).

For cells submitted to the differentiation protocol, they were subjected to the same conditions as the proliferating cells, but after 72 hrs, instead of fixation, the cells were washed twice with differentiation medium in order to remove remaining growth factors and cell debris. Cells were then incubated for a further seven days with no media changes, as previously described ^10^. After seven days, cells were fixed in order to assay cellular markers. Cell markers assessed included the neuronal markers, doublecortin and microtubule-associated protein 2 (MAP2), the astrocyte marker, S100β, and apoptotic marker, CC3. Quantification of immunostaining was performed using the unbiased and semi-automated high-throughput Thermo Scientific Cell-Insight CX5 High Content Screening Platform (Thermo Scientific, Massachusetts, USA). See Supplementary Information, S3-S4 for further details, and Table S1 for a list of primary and secondary antibodies.

**S4: Cell fixing & immunocytochemistry**

At the end of the proliferation/differentiation protocols, the culture medium was aspirated, and cells were washed with warm cell media to remove any cell debris. The cells were then fixed with 50 μl/well of freshly thawed 4% paraformaldehyde (PFA, Alfa Aesar 43368) in phosphate buffered saline (PBS) and incubated for 20 mins at room temperature and in the dark. The PFA was removed after 20 mins and the fixed cells were washed three times with PBS. The plates were then stored in 0.05% Sodium Azide (Sigma, S8032) in PBS, wrapped in parafilm (Sigma, P7793) at 4^O^C.

Immunocytochemistry (ICC) was carried out in order to analyse the fate, viability and health of these cells, based on morphological and protein localisation quantification. For BrdU cells only, following fixation, the cells were incubated with 2N hydrochloric acid for 40 mins in order to denature the DNA strands. Cells were then neutralised with 0.1 M sodium borate buffer for 10 mins and washed with PBS.

For all cells, blocking solution comprised of 5% normal donkey serum (Sigma, D9963) and 0.3% Triton X-100 (Sigma, T9284) in PBS was used to block cells for 1 hr at room temperature. Primary antibodies were then diluted to the appropriate concentration in blocking solution and added to the cells for overnight incubation at 4^O^C. Antibodies were carefully chosen to identify cells at varying degrees of proliferation, differentiation and apoptosis, see *Table 1*. First, the cells were washed with PBS and incubated with blocking solution for 30 mins. During these 30 mins, secondary antibodies were diluted in blocking solution at the appropriate concentrations and added to cells for 2 hrs. Cells were then washed twice with PBS and the nuclei were stained with 300 nM DAPI for 5 mins. Following this, cells were washed twice with PBS and stored in 0.05 % sodium azide in PBS at 4 ^O^C, wrapped in tin foil to block sunlight and prevent photobleaching.

Every plate was designed to contain unstained control cells which were used as a benchmark for background fluorescence in downstream analyses. Antibody combinations were kept the same for all experiments (Supplementary Table 1) and representative images are shown in the main text.

**
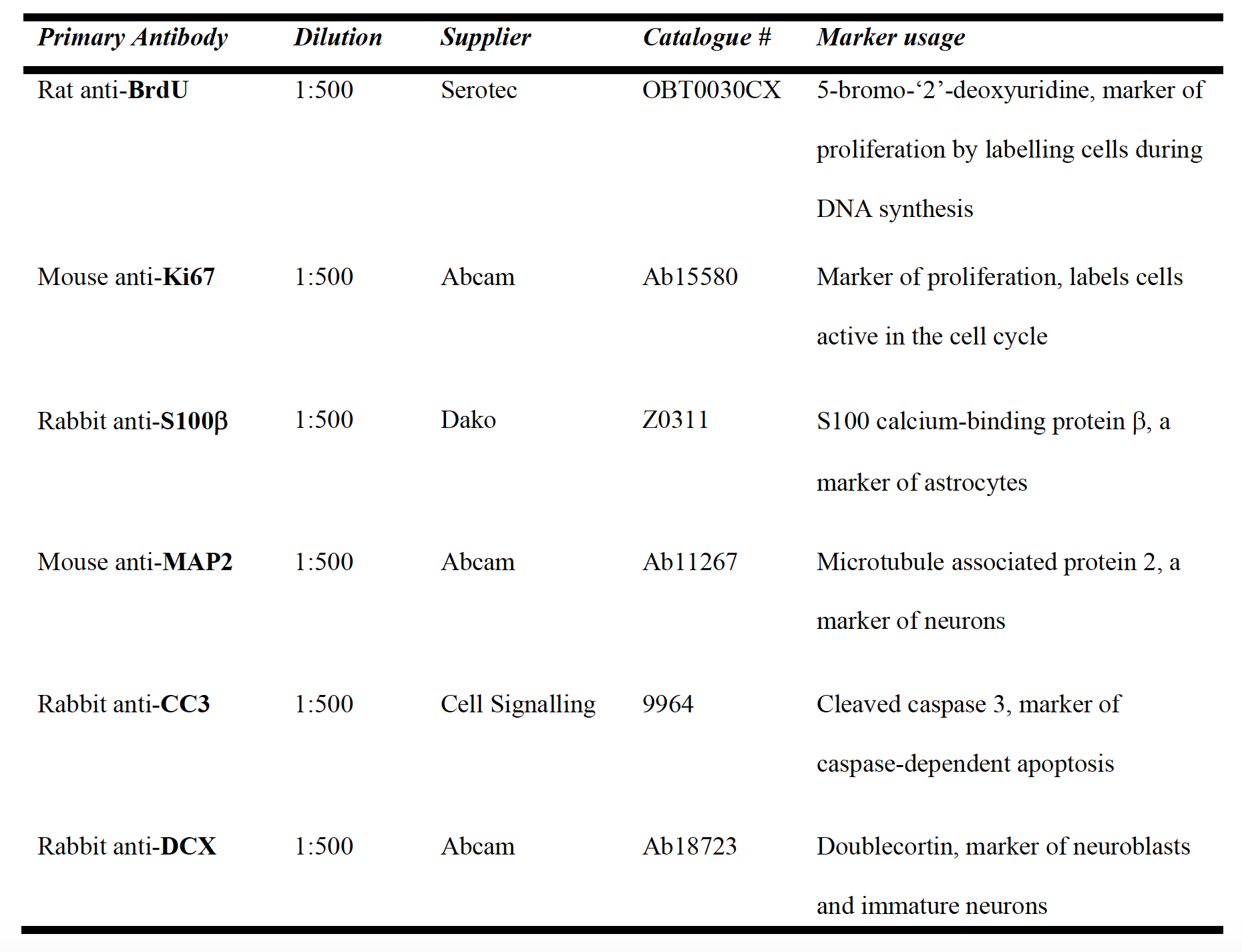
**

**
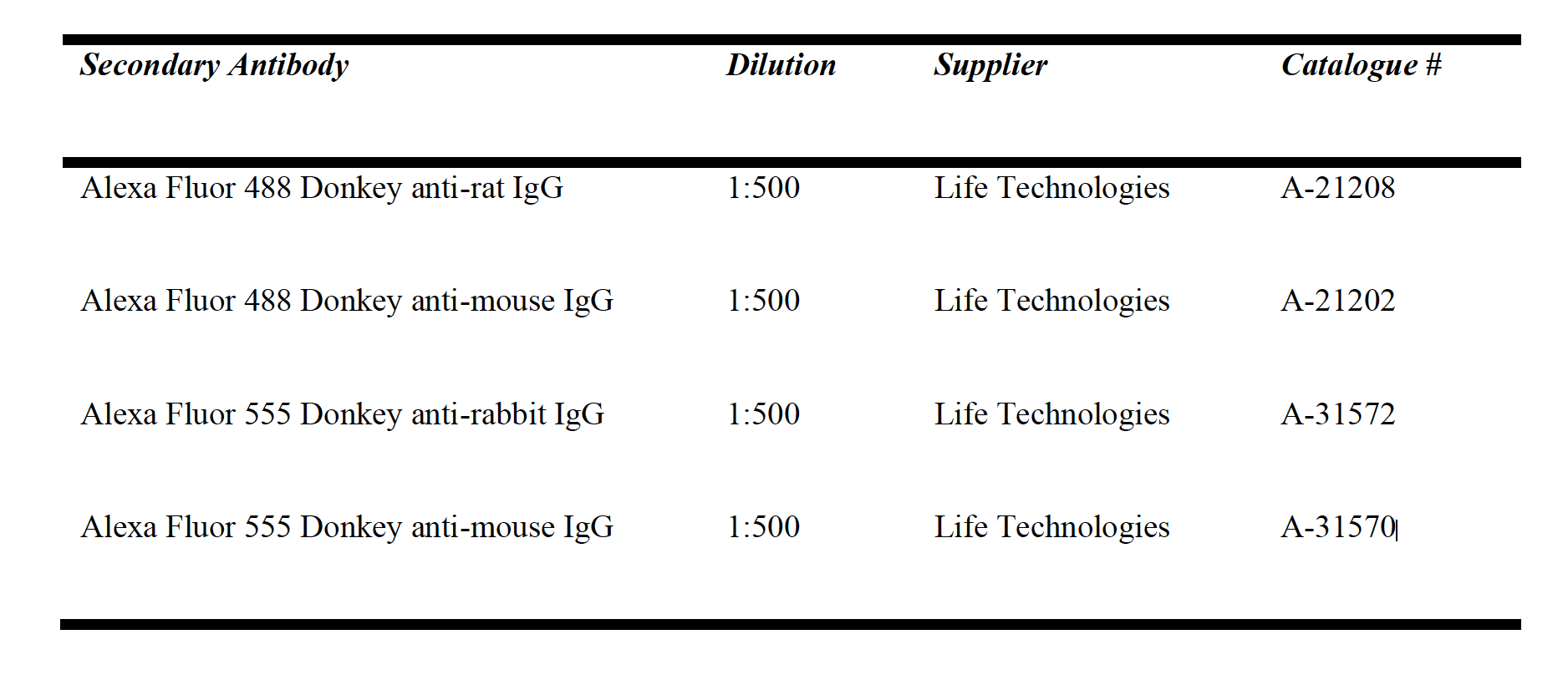
**

**Supplementary Table 1:** Top, a list of primary antibodies used in ICC experiments. Bottom, a list of secondary antibodies used in ICC experiments. For each antibody, there is also information regarding the dilution ratio used in experiments, the supplier, a catalogue number and a summary of how the marker is used (primary antibodies).

**S5: Quantification of cell morphology and protein localisation**

Quantification of cell antibody staining was performed using an unbiased and semi-automated high-throughput Thermo Scientific Cell-Insight CX5 High Content Screening Platform (Thermo Scientific, Massachusetts, USA) alongside the native HCS Studio Cell Analysis Software (Thermo Scientific). Separate protocols were developed for each antibody using the Cell Health Profiling BioApplication, which first identifies cells based on their nuclear staining and then quantifies the intensity of fluorescent staining in user-defined regions. Once a protocol was developed, it was kept constant across all the other plates and biological replicates. On rare occasions the parameters were changed to account for differences in the brightness of immunofluorescence between biological replicates.

**S6: RNA Sequencing Analysis**

Raw reads were downloaded and processed using Trimmomatic 0.38^11^, to prune low quality bases (leading/trailing sequences with phred score < 3, or those with average score < 15 every four bases), or reads below 36 bases in length. Trimmed reads were pseudoaligned to the human reference genome GRCh38 using kallisto^12^. The package tximport^13^ was used to import kallisto output files into DESeq2^14^, summarizing transcript-level information to gene-level expression counts utilizing gene information from the Ensembl Release 99, imported using biomaRt^15^. Differential expression analysis was performed in young versus old cells (N = 4 biological replicates per condition) using the Wald test in DESeq2, controlling for biological replicates. Log2 fold-changes were shrunk using apeglm^16^, and the false discovery rate (FDR) correction was used to control for multiple comparisons. Gene expression differences were considered significant if P_FDR_ < 0.05.

**S7: Effect of chronic high dose lithium treatment on candidate gene expression**

Out of 45 candidates previously identified as being implicated in bipolar disorder or lithium response^17^, only two were significantly affected in our model - Protein kinase B (*AKT1*) and Myristoylated alanine-rich C-kinase substrate *(MARCKS)* (P_FDR_ < 0.1), both of which were downregulated in response to high-dose lithium, Supplementary Figure 1. Protein kinase B (*AKT1*) has previously been identified as a risk factor for bipolar disorder and activates a lithium-responsive cell-survival pathway in mouse kidney cells^18^. Research by Peltier and colleagues suggests that *Akt* also promotes hippocampal cell proliferation and inhibits differentiation^19^. In keeping with this, we find that a downregulation of *AKT1* in our model in response to chronic high-dose lithium, precedes increased cell differentiation. Another gene which shows a decrease in expression in response to high-dose lithium is Myristoylated alanine-rich C-kinase substrate (*MARCKS*). *MARCKS* is a gene that affects neural development and is increased in the platelets of bipolar disorder patients^20^. Our work here supports a possible role for lithium in reversing heightened *MARCKS* expression present amongst bipolar disorder patients.

**Supplementary Figure 1:** A bar chart demonstrating the effect of high dose lithium in our model, on bipolar disorder and lithium response candidate genes. The direction of effect is indicated on the y-axis (log2 fold change, SEM), the gene symbol is indicated on the y-axis, and significant changes (PFDR < 0.05) observed in our cell model are indicated with a *.

**References**

1. Powell TR, Murphy T, Lee SH, Price J, Thuret S, Breen G. Transcriptomic profiling of human hippocampal progenitor cells treated with antidepressants and its application in drug repositioning. *J Psychopharmacol* 2017; **31**(3)**:** 338-345.

2. Powell TR, Murphy T, Lee SH, Duarte RRR, Lee HA, Smeeth D *et al.* Inter-individual variation in genes governing human hippocampal progenitor differentiation in vitro is associated with hippocampal volume in adulthood. *Scientific Reports* 2017; **7**(1)**:** 15112.

3. Powell TR, Murphy T, de Jong S, Lee SH, Tansey KE, Hodgson K *et al.* The genome-wide expression effects of escitalopram and its relationship to neurogenesis, hippocampal volume, and antidepressant response. *Am J Med Genet B Neuropsychiatr Genet* 2017; **174**(4)**:** 427-434.

4. Smeeth DM, Kourouzidou I, Duarte RRR, Powell TR, Thuret S. Prolactin, Estradiol and Testosterone Differentially Impact Human Hippocampal Neurogenesis in an In Vitro Model. *Neuroscience* 2020.

5. Anacker C, Zunszain PA, Cattaneo A, Carvalho LA, Garabedian MJ, Thuret S *et al.* Antidepressants increase human hippocampal neurogenesis by activating the glucocorticoid receptor. *Mol Psychiatry* 2011; **16**(7)**:** 738-750.

6. O'Connell BC, Cheung AF, Simkevich CP, Tam W, Ren X, Mateyak MK *et al.* A Large Scale Genetic Analysis of c-Myc-regulated Gene Expression Patterns * 210. *Journal of Biological Chemistry* 2003; **278**(14)**:** 12563-12573.

7. Palmos AB, Duarte RRR, Smeeth DM, Hedges EC, Nixon DF, Thuret S *et al.* Telomere length and human hippocampal neurogenesis. *Neuropsychopharmacology* 2020; **45**(13)**:** 2239-2247.

8. Severus WE, Kleindienst N, Seemüller F, Frangou S, Möller HJ, Greil W. What is the optimal serum lithium level in the long-term treatment of bipolar disorder--a review? *Bipolar Disord* 2008; **10**(2)**:** 231-237.

9. Powell TR, Powell-Smith G, Haddley K, McGuffin P, Quinn J, Schalkwyk LC *et al.* Mood-stabilizers differentially affect housekeeping gene expression in human cells. *International journal of methods in psychiatric research* 2014; **23**(2)**:** 279-288.

10. Powell TR, Murphy T, Lee SH, Duarte RRR, Lee HA, Smeeth D *et al.* Inter-individual variation in genes governing human hippocampal progenitor differentiation in vitro is associated with hippocampal volume in adulthood. *Scientific Reports* 2017; **7:** 15112.

11. Bolger AM, Lohse M, Usadel B. Trimmomatic: a flexible trimmer for Illumina sequence data. *Bioinformatics (Oxford, England)* 2014; **30**(15)**:** 2114-2120.

12. Bray NL, Pimentel H, Melsted P, Pachter L. Near-optimal probabilistic RNA-seq quantification. *Nat Biotechnol* 2016; **34**(5)**:** 525-527.

13. Soneson C, Love M, Robinson M. Differential analyses for RNA-seq: transcript-level estimates improve gene-level inferences [version 2; peer review: 2 approved]. *F1000Research* 2016; **4**(1521).

14. Love MI, Huber W, Anders S. Moderated estimation of fold change and dispersion for RNA-seq data with DESeq2. *Genome Biol* 2014; **15**(12)**:** 550.

15. Durinck S, Spellman PT, Birney E, Huber W. Mapping identifiers for the integration of genomic datasets with the R/Bioconductor package biomaRt. *Nat Protoc* 2009; **4**(8)**:** 1184-1191.

16. Zhu A, Ibrahim JG, Love MI. Heavy-tailed prior distributions for sequence count data: removing the noise and preserving large differences. *Bioinformatics (Oxford, England)* 2018; **35**(12)**:** 2084-2092.

17. Miranda A, Shekhtman T, McCarthy M, DeModena A, Leckband SG, Kelsoe JR. Study of 45 candidate genes suggests CACNG2 may be associated with lithium response in bipolar disorder. *Journal of affective disorders* 2019; **248:** 175-179.

18. Toyota T, Yamada K, Detera-Wadleigh SD, Yoshikawa T. Analysis of a cluster of polymorphisms in AKT1 gene in bipolar pedigrees: a family-based association study. *Neurosci Lett* 2003; **339**(1)**:** 5-8.

19. Peltier J, O'Neill A, Schaffer DV. PI3K/Akt and CREB regulate adult neural hippocampal progenitor proliferation and differentiation. *Developmental Neurobiology* 2007; **67**(10)**:** 1348-1361.

20. Pandey GN, Dwivedi Y, SridharaRao J, Ren X, Janicak PG, Sharma R. Protein kinase C and phospholipase C activity and expression of their specific isozymes is decreased and expression of MARCKS is increased in platelets of bipolar but not in unipolar patients. *Neuropsychopharmacology* 2002; **26**(2)**:** 216-228.
